# Brief research report pesticide occupational exposure leads to significant inflammatory changes in normal mammary breast tissue

**DOI:** 10.3389/fpubh.2023.1229422

**Published:** 2023-09-14

**Authors:** Ruan Gabriel Soares da Silva, Mariane Okamoto Ferreira, Isabella Mitsu Suo Komori, Henrique Rodrigues Menezes Oliveira, Murilo Galvani Machado, Julia Fernandes Gois Orrutea, Fernanda Mara Alves, Hellen dos Santos Jaques, Janaína Carla da Silva, Janoário Athanazio de Souza, Daniel Rech, Carolina Panis

**Affiliations:** ^1^Laboratory of Tumor Biology, State University of West Paraná, Unioeste, Francisco Beltrão, Paraná, Brazil; ^2^Department of Biochemistry and Molecular Medicine, Université de Montreal, Montreal, Canada; ^3^Francisco Beltrão Cancer Hospital, Francisco Beltrão, Paraná, Brazil

**Keywords:** mammary tissue, inflammation, pesticides, TNF-alpha, oxidative stress

## Abstract

Studies have documented the high occurrence of several tumors, including female breast cancer, in populations occupationally exposed to pesticides worldwide. It is believed that in addition to direct DNA damage, other molecular alterations that indicate genomic instability are associated, such as epigenetic modifications and the production of inflammation mediators. The present study characterized the profile of inflammatory changes in the breast tissue of women without cancer occupationally exposed to pesticides. In samples of normal breast tissue collected during biopsy and evaluated as negative for cancer by a pathologist, oxidative stress levels were assessed as inflammatory markers through measurements of lipoperoxides and total antioxidant capacity of the sample (TRAP) by high-sensitivity chemiluminescence, as well as levels of nitric oxide (NOx) metabolites. The levels of inflammation-modulating transcription factors PPAR-*γ* (peroxisome proliferator-activated receptor gamma) and NF-κB (nuclear factor kappa B) were also quantified, in addition to the pro-inflammatory cytokines tumor necrosis factor-alpha (TNF-*α*) and interleukin 12 (IL-12). The levels of lipoperoxides, TRAP, and NOx were significantly lower in the exposed group. On the other hand, PPAR-*γ* levels were increased in the breast tissue of exposed women, with no variation in NF-κB. There was also a rise of TNF-*α* in exposed women samples without significant variations in IL-12 levels. These findings suggest an inflammatory signature of the breast tissue associated with pesticide exposure, which may trigger mechanisms related to mutations and breast carcinogenesis.

## Introduction

Breast cancer (BC) is the most incident malignant neoplasm that kills women worldwide (1). This pathology is characterized as a multifactorial disease whose development is strongly influenced by intrinsic factors related to the patient’s endocrine aspects and age, as well as extrinsic factors, such as exposure to carcinogenic environmental agents throughout life (2, 3).

Environmental challenges, such as pesticide exposure, have drawn the scientific community’s attention in recent decades because of their carcinogenic potential (4). Due to agricultural feminization, women are continuously exposed to such substances, which can accumulate in the mammary tissue and cause damage (5).

The molecular mechanisms attributable to pesticide carcinogenicity in BC include the generation of inflammatory mediators as oxidative stress and immune response-related molecules, such as cytokines and pro-inflammatory transcription factors (6). Extensive documentation on the damage caused by pesticides to the health of occupationally exposed BC women has been reported. Immune response compromise (7), DNA repair impairment (8) deregulation of estrogen-mediated responses (9) have been described, as well as the high-risk for BC development in rural exposed women (10–13) Human contamination by occasional exposure to pesticides through contaminated water and environment also correlates with the occurrence of breast cancer cases worldwide, further aggravating this scenario (14–18).

Evidence suggests that exposure of non-tumor mammary cells to pesticides results in cancer precursor lesions by altering the expression profile of genes linked to oncogenesis (19).

The mechanisms described include increased expression of the tumor necrosis factor-1 receptor (20), DNA damage with changes in estrogen pathways and cell proliferation (21); changes in breast tissue development (22) and epigenetic changes (23), among other effects.

Despite this, little is known about the mechanisms involved in human mammary carcinogenesis in this setting. The impact of pesticides on the normal breast tissue of occupationally exposed women is unknown. The available studies are limited to mechanistic investigations *in vitro* or experimental data. Thus, the present work focused on investigating the impact of chronic and continuous occupational exposure to pesticides on inflammatory markers in female farmers’ breast tissue. We characterized immune and oxidative stress mediators potentially impacted by pesticide exposure that might configure an inflammatory signature in a Brazilian rural worker population.

## Methods

### Study design

**Figure 1:**
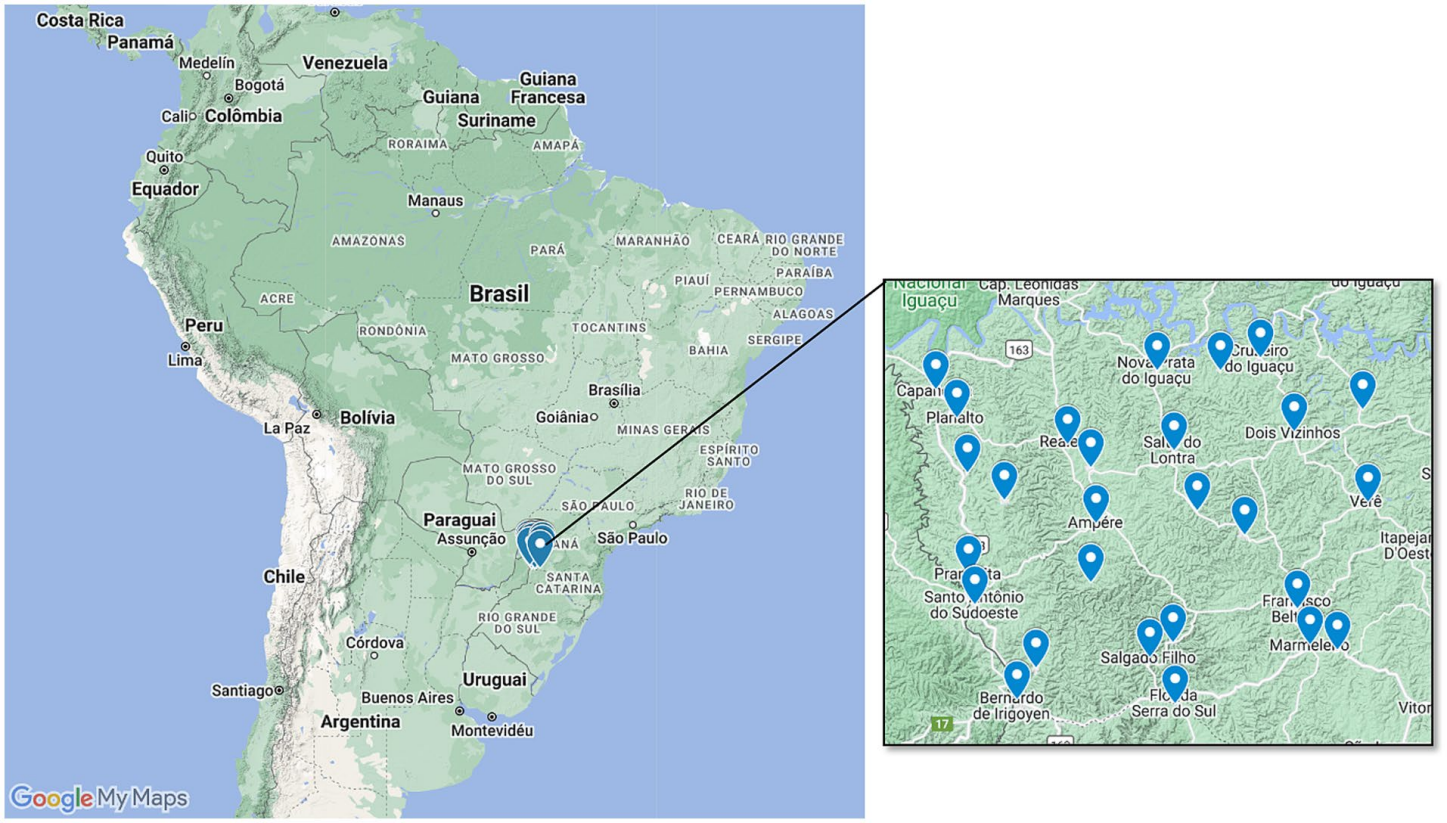
Geographic representation of the study area. We included women who attended the 8th Health Regional of Paraná state, which aggregates 27 municipalities mainly characterized by agriculture and rural work.

This case–control cohort study was submitted to the Institutional Human Research Ethics Committee registered under CAAE 35524814.4.0000.0107. All participants signed consent terms. All patients referred for a surgical procedure with suspicious breast lesions assisted by Francisco Beltrão Cancer Hospital (Ceonc) at the 8^th^ Health Regional of Paraná state from May 2015 to August 2022 were screened (*n* = 602). The study included women from 27 municipalities (Figure 1) characterized for high use of pesticides and predominant rural work. We chose to study this region because Paraná is among the five states that sell the most pesticides in the country. Agricultural production has a significant share in the composition of the Gross Domestic Product (GDP) of the 27 municipalities that comprise the Southwest of the state, characterized by the extensive use of pesticides. In this area, more than 50% of the population is engaged in agricultural activities, focusing on family farming and extensive occupational exposure to pesticides used in monocultures such as soy, corn, and wheat.

As shown in Table 1, almost all the municipalities that compose the 8th Health Regional have a significant pesticide trade over the Brazilian average, estimated as about 6 kg/*per capita*. Also, these cities have high volumes for pesticide trade in tons and show an expressive number of individuals at risk. The main pesticides reported as used in their farms were glyphosate, atrazine, and 2,4D in almost 60% of the crops. All patients included in the study were living in one of the 27 municipalities studied.

**Table 1:** Sociodemographic and pesticide consumption characteristics of the study population.

Municipality	Mean volume of pesticides traded (2011–2016, tons)	Pesticide consumption (tons *per capita*)	Number of individuals at risk
Ampere	126,0	6,0	19,466
Bela Vista da Caroba	56,8	13,0	3,404
Bom Jesus do Sul	24,9	5,0	3,472
Capanema	255,8	13,0	19,172
Coronel Vivida	518,8	24,0	20,430
Cruzeiro do Iguaçu	100,9	21,0	4,229
Dois Vizinhos	374,6	8	41,424
Enéas Marques	48,7	8	5,906
Flor da Serra do Sul	68,3	15,0	4,583
Francisco Beltrão	341,4	3,4	93,308
Manfrinópolis	341,4	4,0	2,442
Marmeleiro	162,6	12,0	14,407
Nova Esperança do Sudoeste	36,3	5,7	5,014
Nova Prata do Iguaçu	250,0	19,0	10,540
Pérola do Oeste	48,7	22,0	6,232
Pinhal de São Bento	31,1	13,0	2,742
Planalto	192,9	15,0	13,385
Pranchita	232,9	39,0	5,035
Realeza	236,7	22,0	16,976
Renascença	339,2	49,0	6,772
Salgado Filho	34,2	7,0	3,389
Salto do Lontra	185,7	12,0	14,957
Santa Izabel do Oeste	246,5	32,0	14,924
Santo Antônio do Sudoeste	173,6	8,0	20,354
São Jorge D’Oeste	377,4	40,0	9,005
Verê	366,0	41,0	7,094

Based on the analysis of the biopsies, only the patients whose result was a benign sample entered the study. An instrument validated for this purpose was further used to characterize occupational exposure to pesticides (24). The exposure criteria were based on continuous, unprotected, and direct handling of pesticides. Rural women with a history of direct handling of pesticides without wearing protective gloves during the preparation and dilution of the poison solution, application of pesticides, and/or decontamination of personal protective equipment (PPE) and/or washing of clothes used during spraying, and that reported living at least 50% of their lives under direct pesticide handling at least twice a week during all weeks of the year were considered exposed. The unexposed group consisted of urban female workers with no previous or current history of occupational exposure to pesticides. Based on data about pesticide exposure and inclusion criteria, 102 healthy women were divided into occupationally exposed to pesticides (*n* = 42) or not occupationally exposed to pesticides (*n* = 60).

### Sample obtention and processing

Fragments of breast tissue samples were obtained during biopsy surgery for diagnosis and subsequently frozen for analysis. Tissue fragments were homogenized in sterile saline phosphate buffer 10 mM pH 7.4 with a grinder to obtain a homogenate at a final concentration of 50 mg/mL. All methods described used this concentration of tissue homogenate, and all measurements were performed on the same day of sample processing.

### Oxidative stress evaluation

For lipoperoxides, 200 μL aliquots of tissue homogenate (50 mg/mL) were added to 20 μL of 3 mM t-butyl solution. Readings were performed in a Glomax luminometer (Glomax, Promega). The results were expressed in relative light unities (RLU), and the entire profile of the curve was used as an indicator of lipid peroxidation. To determine the total antioxidant capacity of the samples (TRAP), breast tissue homogenates (50 mg/mL) were added to a reaction medium consisting of 20 mM 2,2′-azobis (2-amidinopropane) (ABAP) and 40 μM luminol. ABAP is a source of free radicals that degrades at temperatures above 28°C and reacts with luminol present in the medium, producing photons detected by chemiluminescence. The addition of a diluted sample inhibits the ABAP degradation reaction for a period (induction time) and is directly proportional to the plasma concentration of TRAP antioxidants. For the calculation of TRAP, the induction time of the sample (the time during which the antioxidants in the sample can inhibit the action of ABAP) was compared to that of the standard antioxidant (Trolox) and expressed in μM Trolox/g tissue (25). TRAP was analyzed by high-sensitivity chemiluminescence in a Glomax 20/20 luminometer (Promega, United States). To measure the levels of nitric oxide (NOx) metabolites, the method of converting nitrate to nitrite was used by the cadmium-copper reaction, and the detection of total nitrite was by the Griess method (26). Absorbance was read at 550 nm using a standard microplate reader. Results were expressed in μM NOx/mg of tissue.

### Evaluation of transcription factors PPAR-*γ* and NFκb activity, and quantification of cytokine levels

Commercial enzyme immunoassay kits (Cayman Chemical, United States) were used to analyze the levels of transcription factors in breast tissue homogenates. The kit detects nuclear PPAR-*γ* and NF-kB present in tissue homogenates. To evaluate the levels of cytokines in breast tissue homogenates, commercial enzyme immunoassay kits (Invitrogen, United States) were used to quantify TNF-*α* and IL-12 cytokines.

### Data analysis

Data distribution was tested using the Shapiro–Wilk test. Thus, variables with normal distribution were analyzed using parametric tests. When the assumption of normality was not met, non-parametric tests were used. Student’s t-test or Mann–Whitney test was used to compare data between exposed and unexposed groups. The results were analyzed using GraphPad Prism version 9.0 (Graphpad Software, San Diego, CA, United States). In the results, parametric data are described as mean ± standard error, and non-parametric results are presented as medians. For frequency analyzes, Fisher’s exact test was used. A value of *p* < 0.05 was considered significant.

## Results

Table 2 presents data on menopause, age, and BMI of the 102 women included in this study, divided into groups exposed and not exposed to pesticides. Since such parameters are known factors that can affect the inflammatory profile, we collected this information and compared both groups to ensure they were not different in this concern, aiming to reduce critical confounding factors. As for the exposed group, 26.2% were over 50 years old at diagnosis, 35.7% were in menopause at the time of collection, and 54.8% had BMI classified as overweight/obese. In the group of unexposed women, 25% were over 50 years old at diagnosis, 28.3% were in menopause, and 53.3% had overweight/obese BMI.

**Table 2:** Age at diagnosis, menopausal status at diagnosis, and body mass index (BMI) of women in the study distributed according to their pesticide occupational exposure profile.

		Exposed (%)	Unexposed (%)	*p*-value (Fisher)
Age at diagnosis	≥50 y	26.2	25	>0.9999
	<50 y	73.8	75	
Menopausal status	Yes	35.7	28.3	0.2886
	No	64.3	71.7	
BMI	≥25 kg/m^2^	54.8	53.3	>0.9999
	<25 kg/m^2^	45.2	46.7	

**Figure 2:**
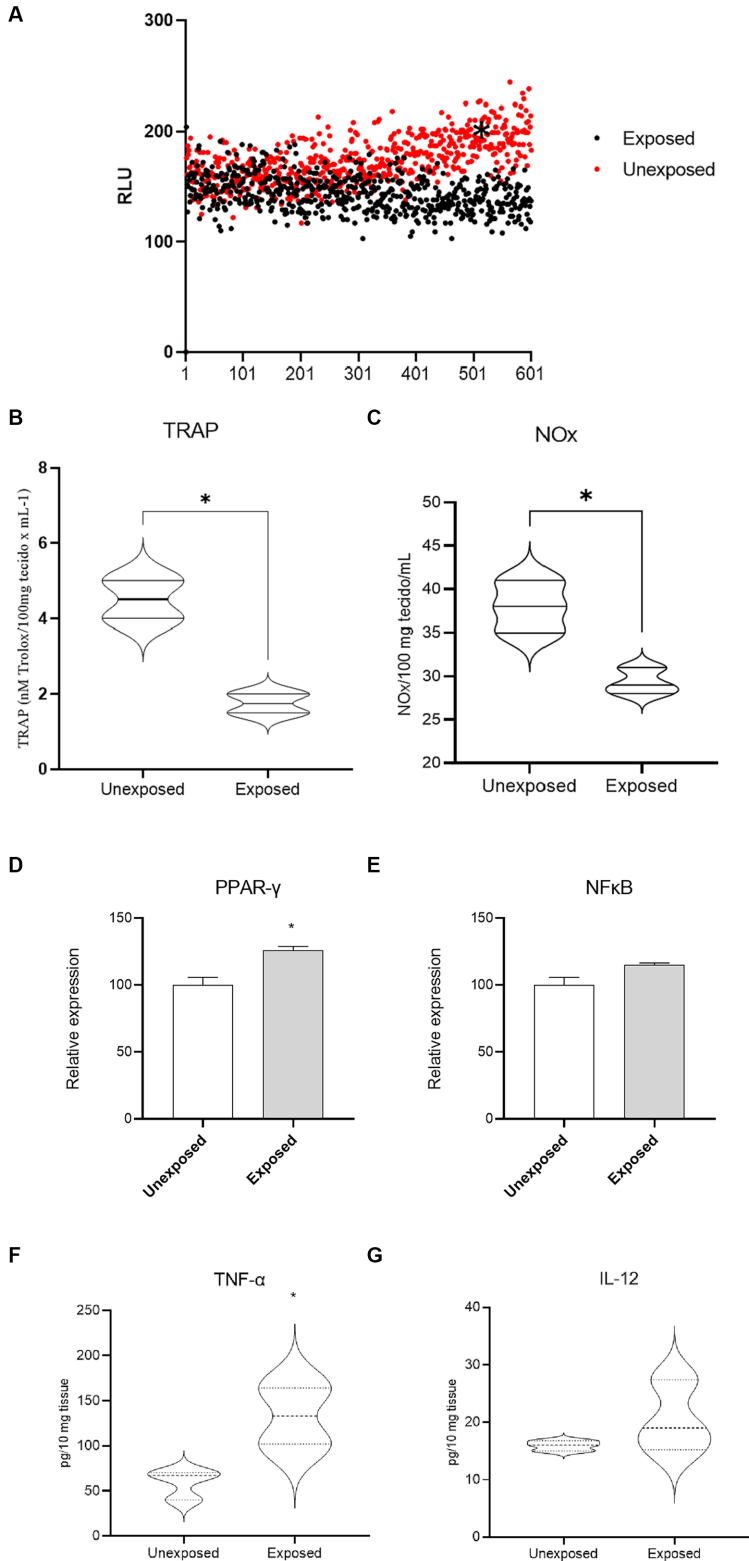
Breast tissue oxidative stress profile of women occupationally exposed or not to pesticides. In **(A)**, levels of lipoperoxides, in **(B)**, TRAP and in **(C)**, levels of NOx metabolites, transcription factors PPAR-*γ* **(D)** and NFκB **(E)**, AND TNF-*α* **(F)** and IL-12 **(G)**; * indicates *p* < 0.05; RLU = relative units of light.

To characterize the oxidative stress profile of the analyzed samples, the levels of pro-oxidants (lipoperoxides and NOx) and antioxidants (TRAP) were evaluated by high-sensitivity chemiluminescence. As shown in Figure 2, lipoperoxide levels were significantly higher in the breast tissue of women not exposed to pesticides (Figure 2A, median of 143 RLU for exposed women and 172 RLU for non-exposed women, *p* < 0.0001). On the other hand, the antioxidant capacity, assessed by TRAP (Figure 2B), was significantly reduced in those exposed compared to those not exposed (4.5 ± 0.5 nM Trolox and 1.75 ± 0.25 nM Trolox, respectively, *p* = 0.0389). NOx levels (Figure 2C) were also reduced in samples of women exposed to pesticides compared to non-exposed women (38.00 ± 1.73 μM and 29.33 ± 0.88 μM respectively, *p* = 0.0112).

Levels of the transcription factor PPAR-*γ* (Figure 2D) were increased in breast tissue samples from women occupationally exposed to pesticides when compared to those not exposed (relative expression increased by 26%, *p* = 0.0283). In regarding NF-κB levels (Figure 2E), no significant variation was observed (*p* = 0.0679). The evaluation of the cytokine profile in breast tissue revealed an increase in TNF-alpha in samples from occupationally exposed women compared to non-exposed ones (Figure 2F, 137.1 ± 18.34 pg./mL and 59.01 ± 9.53 pg./mL respectively, *p* = 0.0196). No significant variations were observed in IL-12 levels (Figure 2G, *p* > 0.05).

## Discussion

This study evaluated the inflammatory profile of non-cancerous breast tissue of healthy agricultural women occupationally exposed to pesticides by analyzing tissue oxidative stress markers, cytokines, and transcription factors. We observed that pesticide exposure induces significant inflammatory changes in normal breast tissue, providing an environment of sustained inflammation with the expression of anti-tumor defense mechanisms, which were not observed in samples from non-exposed women. Here we describe alterations induced by chronic and continuous exposure to pesticides, showing altered mechanisms known to generate breast cancer in the normal mammary tissue.

Few studies have focused on understanding the changes that precede breast cancer, and there is little evidence of the changes in normal breast tissue. Most studies focus on understanding the systemic changes in this context, mainly reporting data on blood changes (9, 24, 27).

It is not clear the exact mechanisms that can lead a normal mammary cell to turn into breast cancer cells, but evidence point out pathways linked to inflammation (19). The tumor-promoting inflammation is a hallmark of cancer that enhances tumorigenesis and progression. Paradoxically, it is driven by cells and mediators derived from the immune system (6, 28). Tumor-associated inflammatory response modulates other tumor-promoting events such as providing growth factors, sustaining the replicative signaling, enabling angiogenesis, invasion, and metastasis, and supporting genomic instability by oxidative stress production (29). Further, inflammation has been pointed out as a phenomenon that occurs in the early stages of cancer, fostering early-stage tumors to progress (30).

Pesticide exposure increases the risk of developing breast cancer, as evidenced by increased oxidative stress biomarkers, causing direct DNA damage and high mutational load (8, 31, 32). Therefore, pesticides support a tumor-enabling environment. Since pesticides are known as potentially, probably, and/or proven carcinogens, we hypothesized that women chronically exposed to such substances could have changes in the inflammatory profile of their mammary tissue detected before any cancerous manifestation. To answer this question, we selected a group of rural women that reported continuous exposure during their lifetime by manipulating, applying, or decontaminating equipment containing pesticides (named as exposed). We compared it to women that had never been in contact with pesticides (named as unexposed).

The comparative analysis of the mammary tissue from both groups revealed that pesticide-exposed women lacked antioxidant defenses associated with reduced levels of nitric oxide metabolites. Antioxidant impairment is frequently reported in cancer as a reaction to oxidative/nitrosative stress generation (33). It, in our case, may indicate that the breast tissue from pesticide-exposed women is mobilizing its antioxidant defenses against the continuous pesticide-induced oxidative/nitrosative environment. The changes in the production of oxidative/nitrosative stress mediators found here in normal breast tissue deserve attention because it is directly associated with the development and progression of breast cancer (34, 35) and the occurrence of worse prognostic outcomes (7, 8, 36).

Since oxidative stress changes are frequently associated with cytokine production, we investigated the mammary tissue profile of some essential cytokines and transcription factors linked to oxidative/nitrosative stress and cancer. We observed a significant increase in the mammary expression levels of TNF-*α* in pesticide-exposed women compared to the unexposed ones. Cellular neoplastic transformation is predisposed by various chronic inflammatory conditions, primarily injury, repair, and resolution. The inflammatory process is mediated by messenger molecules such as cytokines, prostaglandins, chemokines, and angiogenic factors (33). Activation of transcription factors through TNF-*α* regulates gene expression in cell proliferation, apoptosis, and carcinogenesis (37). Thus, the augmented TNF-*α* levels may disturb tissue homeostasis in chronic pesticide exposures.

Interestingly, no differences were found regarding NFκB expression, a major transcription factor related to inflammation. Because of this, we further investigated the expression of another transcription factor related to inflammatory responses and triggered by oxidative/nitrosative stress (38), the proliferator-activated receptor *γ* (PPAR-*γ*). The overexpression of PPAR-y has an anti-tumor effect and negatively modulates other transcription factors, such as NFkB (39–42). Here we observed increased expression of PPAR-y in the breast tissue of women exposed to pesticides compared to those not, suggesting that pesticides are essential aggressors of the antioxidant system (as we demonstrate by tissue TRAP consumption) in the normal mammary gland.

Collectively, these results indicate a putative precancerous mechanism triggered by chronic and severe pesticide exposure in the mammary tissue, driven by antioxidant depletion inducing PPAR-*γ* overexpression and TNF-*α* production. Considering the protective effect of antioxidants against cancer and the pro-tumor effects of TNF-*α*, this scenario represents a disbalance that can stimulate cell proliferation, DNA damage, and immune deregulation, favoring BC development. It is important to highlight that pesticides, including those reported here in our study area, are pointed out as endocrine disruptors (43–46), an event intrinsically linked to deregulated immunological and inflammatory responses (9) that could confer some carcinogenic potential to such substances. Our findings strengthen several worldwide studies demonstrating augmented breast cancer risk in pesticide occupationally exposed women (47–55) by adding information about possibly implicated mechanisms in the pre-carcinogenic stages. Detecting pesticides in urine, blood, breast milk, and mammary tissue samples from exposed women reinforces the idea that they may have a systemic impact (14, 56–58).

This study has limitations, including the modest sample size, the need for measurements of the markers evaluated at more collection points, and the absence of pesticides residues measurement. Also, other risk factors not assessed in the study, such as dietary habits and lifestyle, could affect the results. However, as our focus was on breast tissue analysis, this temporal evaluation of markers would be unfeasible due to the invasiveness of the sample collection method. The strong point of this study is the evaluation of such phenomena in breast tissue samples, which is reported in very few studies and has never been reported in the context of pesticides as far as we know.

Since pesticide exposure has been linked to breast cancer risk worldwide (10–13, 59), our study adds evidence concerning which mechanisms are putatively triggered before the pre-cancerous stages. We characterized a scenario of sustained inflammation in women subjected to chronic exposure to pesticides, with the production of TNF-alpha, activation of PPAR-*γ*, and consumption of tissue antioxidant defenses, which configure important mechanisms that could be involved with pre-cancerous lesions and breast carcinogenesis under sustained conditions. Such findings may draw attention, especially in regions with severe pesticide exposure, such as Brazil (7, 9, 17, 24), where women are a silent and significant part of the labor in agriculture.

## Data availability statement

The raw data supporting the conclusions of this article will be made available by the authors, without undue reservation.

## Ethics statement

The studies involving humans were approved by Universidade Estadual do Oeste do Paraná Ethics Comittee. The studies were conducted in accordance with the local legislation and institutional requirements. The participants provided their written informed consent to participate in this study.

## Author contributions

RS, MF, IK, HO, MM, JO, FA, HS, JCS, JAS, DR, and CP contributed to the study conception and design, material preparation, data collection, and analysis. The first draft of the manuscript was written by RS and CP. All authors contributed to the article and approved the submitted version.

## Funding

This work was supported by Araucária Foundation (Fundação Araucária) (call 048/2021), Research Program for SUS – PPSUS (Programa de Pesquisa Para o SUS – PPSUS), Coordination for the Improvement of Higher Education Personnel (Coordenação de Aperfeiçoamento de Pessoal de Nível Superior, CAPES), National Council for Scientific and Technological Development – CNPq, Universal Grant (Conselho Nacional de Desenvolvimento Científico e Tecnológico – CNPq, Edital Universal) (Grant numbers 402364/2021-0 and 305335/2021-9).

## Conflict of interest

The authors declare that the research was conducted in the absence of any commercial or financial relationships that could be construed as a potential conflict of interest.

## Publisher’s note

All claims expressed in this article are solely those of the authors and do not necessarily represent those of their affiliated organizations, or those of the publisher, the editors and the reviewers. Any product that may be evaluated in this article, or claim that may be made by its manufacturer, is not guaranteed or endorsed by the publisher.
